# The regulatory governance conditions that lead to food policies achieving improvements in population nutrition outcomes: a qualitative comparative analysis

**DOI:** 10.1017/S1368980021004730

**Published:** 2022-05

**Authors:** Yandisa Ngqangashe, Sharon Friel, Ashley Schram

**Affiliations:** Menzies Centre for Health Governance, School of Regulation and Global Governance, Coombs Extension Building 8, Fellows Road, Australian National University, Acton, 2601 Canberra, Australia

**Keywords:** Qualitative comparative analysis, Nutrition, Food policy, Governance, Regulation, Outcomes

## Abstract

**Objective:**

To identify the regulatory governance factors that lead to food policies achieving improvements in food environment, consumer behaviour and diet-related health outcomes.

**Design:**

Qualitative comparative analysis (QCA) was used to investigate the relationship between regulatory governance conditions and population nutrition outcomes. The regulatory governance conditions examined entailed: high industry involvement in the policy process, regulatory design, policy instrument design, policy monitoring and enforcement.

**Participants:**

*n* 29 policy cases in the policy areas of food reformulation, nutrition labelling, food taxation and food marketing.

**Setting:**

Policies implemented in thirteen countries.

**Results:**

Comprehensive monitoring was identified as a *necessary* regulatory governance condition for food policies to have an impact and was present in 94 % of policy cases that had a positive impact on nutrition outcomes. We identified two *sufficient* combinations of regulatory governance conditions. The first *sufficient* combination of conditions comprised an absence of high industry involvement in the policy process, combined with the presence of strict regulatory design, best-practice instrument design, and comprehensive monitoring and enforcement. Ninety-six percent of policy cases with positive impacts on nutrition outcomes displayed this combination. The second *sufficient* combination of conditions comprised an absensce of high industry involvement in the policy process, best practice instrument design and comprehensive monitoring. Eighty-two percent of policy cases with positive impacts on nutrition outcomes displayed this combination.

**Conclusion:**

These findings show the importance of regulatory governance on policy outcomes. They suggest a need for more government-led nutrition policy processes and transparent monitoring systems that are independent from industry.

The development and implementation of policy actions to promote healthy diets is part of the WHO Global Strategy on Diets, Physical Activity and Health^(1)^. These actions include policies that regulate the supply and availability of food, including food composition and how food is labelled, promoted and priced^(2–4)^. There are efforts to track country progress on the adoption and implementation of food policies through initiatives such as the World Cancer Research Fund International NOURISHING framework^(2)^ and the International Network for Food and Obesity Research, Monitoring, and Action Support (INFORMAS)^(4)^. However, the reported efficacy and effectiveness of the policies and their associated regulatory governance varies across jurisdictions^(5–7)^. This study seeks to understand why certain policies work and others fail focusing on regulatory governance.

Regulatory governance entails multiple actors including governments, industry, civil society and consumers, as well as institutional processes and political contexts within which policies are developed, designed and implemented^(8)^. Guidi et al. (2020) posit that the implementation of policies and policy outcomes are a result of how the policies are designed during the formulation stage and the design of the policy itself is shaped by the agenda setting stage of the policy^(9)^. In line with this position, this study considers actors, contexts and processes (regulatory governance) across the policy cycle, from agenda setting, formulation through to implementation. Agenda setting focuses on the point in the policy process where various issues are competing for policy attention and how the issues are problematised^(10)^. The formulation stage examines the process of outlining the policy objectives and choosing instruments to effect the objectives, while implementation covers the stage of resourcing and actualising the policies over time^(10)^.

Research on regulatory governance of population nutrition policies shows that the agenda setting stage of food policies is shaped by powerful actors involved with the issue and how they frame the issues, and political contexts and existence of evidence^(11–13)^. The work of Magnusson and Reeve identified various shortcomings with the regulatory governance of the formulation and implementation stages of population nutrition policies^(14–17)^. These include lack of transparency and accountability of pure industry self-regulation and public–private partnerships, the use of poor design standards and lack of monitoring and enforcement^(15–17)^. While this literature outlines the regulatory governance of food policies at different stages of the policy cycle, it does not identify how regulatory governance conditions across the policy cycle influence food policy outcomes.

Our study seeks to provide a comprehensive empirical examination of the relationship between the regulatory governance of the policy cycle and policy outcomes. Thus, the aim of this study is to identify the regulatory governance factors that lead to food policies achieving improvements in outcomes related to the food environment, consumer dietary behaviour and diet-related health.

## Methods

### The qualitative comparative analysis approach

In this study, we investigate which regulatory governance factors across the policy cycle support improvements in population nutrition outcomes. We employed an innovative methodology – qualitative comparative analysis (QCA). QCA is helpful for understanding the varying levels of efficacy of food policies and for improving the regulatory governance of current and future policies, thereby enabling better outcomes for population nutrition. QCA analyses causality by combining a comparative case-based approach, often used in qualitative research, with mathematical approaches used in quantitative research^(18,19)^. Using QCA has a number of advantages: first, it enables identification of more than one causal pathway to an outcome (equifinality). Second, QCA enables analysis of different factors that lead to the same outcome (conjectural causation). Third, QCA allows causal asymmetry whereby the presence of conditions that lead to success does not mean their absence leads to failure. Last, QCA can identify causal conditions that lead to different outcomes (multifinality)^(20,21)^. This broad approach to causality is necessary for understanding the relationship between regulatory governance and nutrition policy because food policies are developed, adopted and implemented in complex contexts and processes^(12,22)^. The QCA approach therefore enables an examination of how these contexts and processes across different policy cases produce or combine to produce improvements in food environments, consumer behaviours and diet-related health outcomes.

### Applying qualitative comparative analysis in this study

**Fig. 1:**
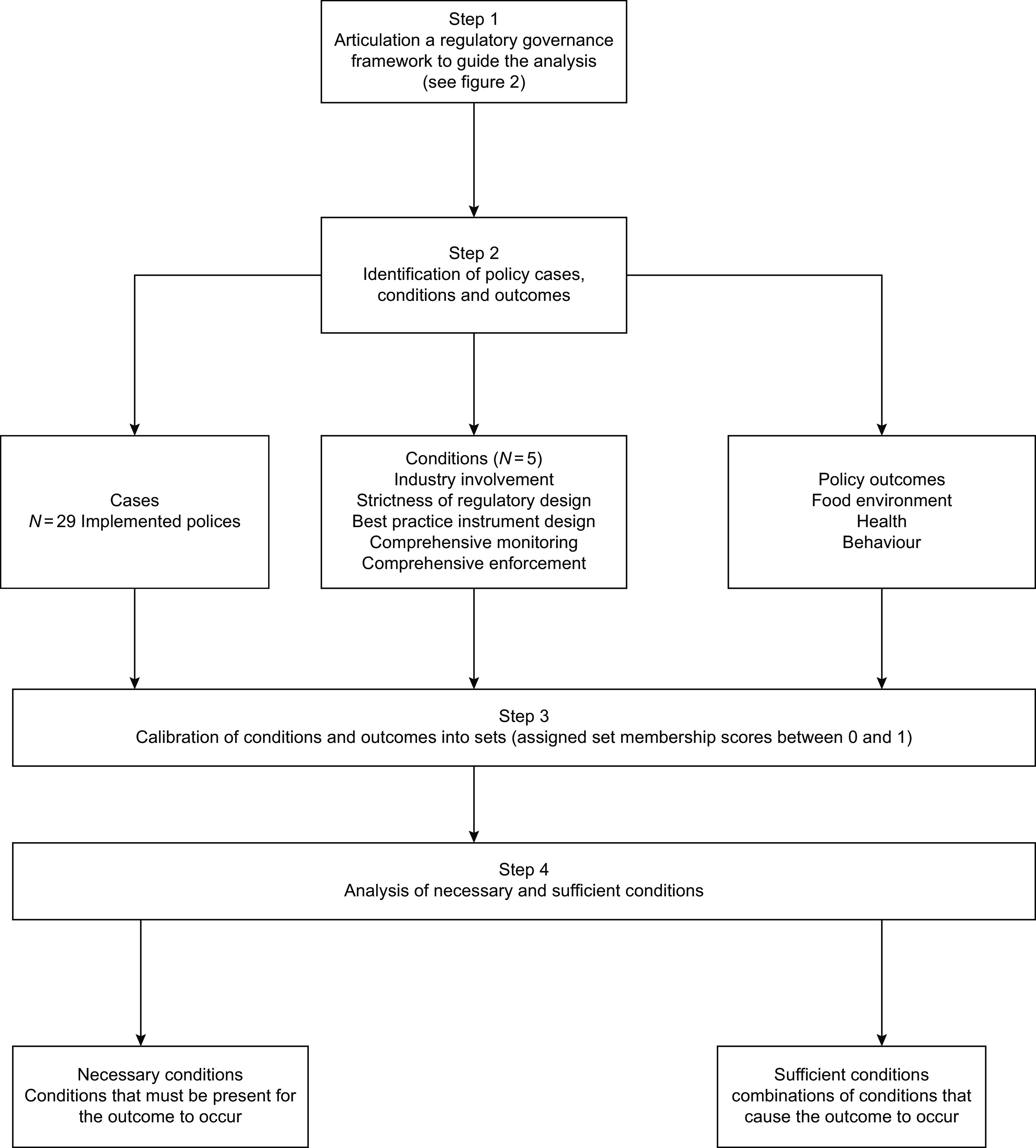
QCA analysis process

We applied QCA in four steps. First, we articulated a theory-informed regulatory governance analytical framework to guide the identification of regulatory governance factors to include in the analysis. Second, using the framework, we conducted a qualitative analysis of secondary data to identify policy cases, conditions and outcomes for the QCA analysis. Third, we assigned set membership scores to the regulatory governance conditions and policy outcomes. Lastly, we analysed the data using a QCA data analysis program (fs/QCA) http://www.socsci.uci.edu/∼cragin/fsQCA/software.shtml to determine the regulatory governance conditions for achieving improvements in food policy outcomes (food environment, dietary behaviours and health). Each step is now explained in detail and presented in Fig. 1.

### Step 1: Articulating the regulatory governance analytical framework

**Fig. 2:**
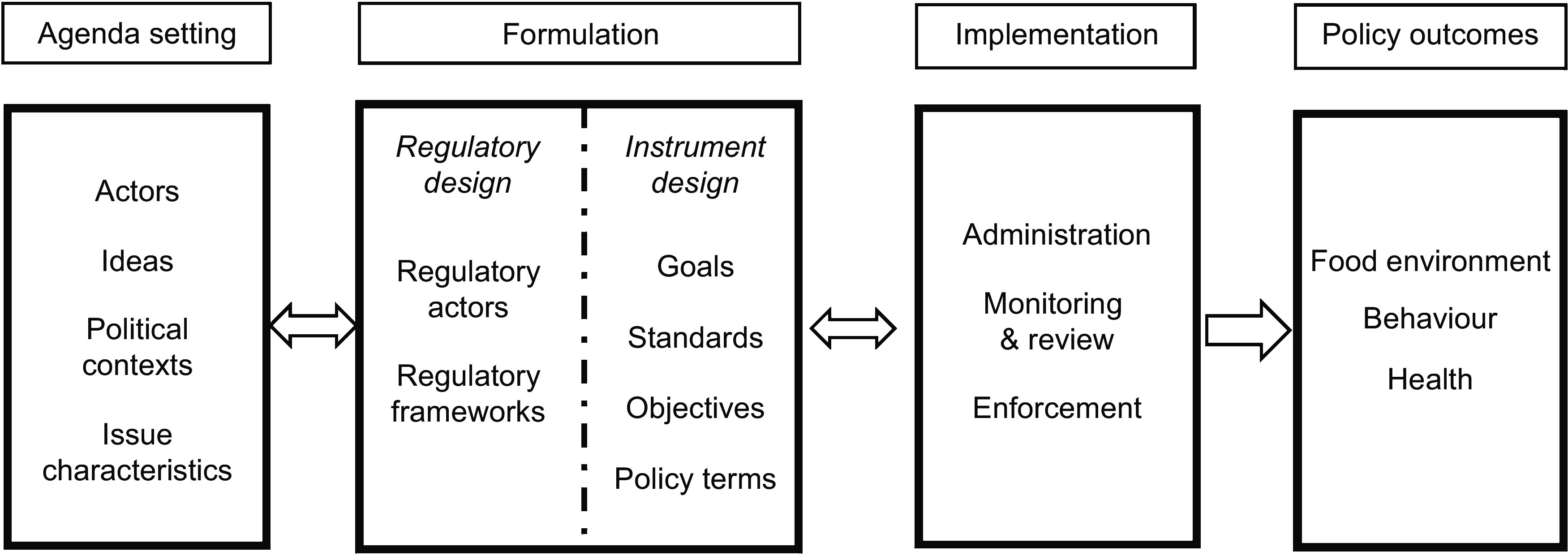
Analytical framework

The theory-informed analytical framework has four components, organised according to the policy cycle (agenda setting, formulation and implementation) and policy outcomes (see Fig. 2).

Agenda setting refers to how (food) policy agendas are decided and shaped. We drew on Shiffman and Smith’s work on the determinants of political priority, which identifies the role of different actors, ideas and institutional and political contexts in shaping the adoption and formulation of policies^(23)^.

The policy formulation stage of the framework has two components: regulatory design and instrument design. Regulatory design is concerned with the form of policy action – combinations of actors involved in the development and implementation of policies – as well as the regulatory frameworks that underpin the policies. Here we draw on the theory of responsive regulation that emphasises that there are networks of actors (government, industry and civil society) and diverse regulatory frameworks involved in rule making, monitoring and enforcing policies^(24)^. These include government regulation through laws such as taxes and co-regulation where the government delegates aspects of rulemaking, monitoring or enforcement to statutory regulatory authorities and/or the industry as well as non-legislative approaches such as public–private partnerships between the governments, civil society and pure self-regulation^(24)^.

Instrument design relates to the purpose of the policy, design of the selected policy instrument as well as the substantive terms and conditions of the policy^(14)^. The relevant factors for instrument design were informed by the regulatory scaffolding framework for ensuring accountability and transparency of policies, which describes four essential design elements: goals, standards, objectives and terms^(14)^.

The policy implementation stage is concerned with the actors, institutions and the processes used to administer, monitor and enforce the policies. These factors were also identified using the regulatory scaffolding framework^(14)^ and includes strengthening administration, monitoring and enforcement of policies^(14)^.

Finally, policy outcomes were defined as any improvements to the food environment, diet-related behaviour or diet-related health of populations that occurred after the policy was implemented.

### Step 2: Identifying policy cases, conditions and outcomes

#### Policy cases

In our analysis, policy cases entailed policies in the areas of food reformulation, nutrition labelling, food marketing and taxation that were implemented by government, industry or through public–private partnerships. These policies could include legislation or partnerships between governments and the industry or voluntary actions by the industry. These policies seek to steer industry and consumer behaviour towards food environments that support the supply and consumption of affordable healthy food options and are part of the WHO Global Action Plan for the Prevention and Control of non-communicable disease^(25)^. Labelling, marketing, taxation and reformulation policies have gained significant policy attention at the global and national levels, hence we thought prudent and practical to focus on these. In 2019, we conducted a narrative review of international peer-reviewed and grey literature on regulatory governance of implemented food policies (*n* 73) and on policy outcomes (*n* 224)^(13)^. A year after the initial review, we conducted an additional literature search to identify any studies published between August 2019 and August 2020 followed by an additional grey literature search to fill gaps in policy cases that had insufficient information on one or more regulatory governance conditions. Records that were based on policy cases that did not have enough information on all the regulatory conditions were excluded. The final conditions and policy outcomes were extracted from 147 records that had information on regulatory governance and policy outcomes of twenty-nine policy cases. The final list of policy cases included in the QCA analysis had information on the regulatory governance across stages of the policy cycle and information on the impact of that policy on outcomes (either the food environment, behaviour or health outcomes). The list of included policy cases and literature sources is shown in Supplemental File 1.

### Conditions

The regulatory governance factors identified through the review were qualitative descriptions of the policy process such as the political contexts that shaped the adoption and design of the policies, the different regulatory designs and how the policies were monitored and enforced. In QCA theoretical concepts such as these regulatory governance factors we identified in the review are operationalised as ‘conditions’. The process of operationalisation entails translating broad concepts into measurable variables. Below we explain how we selected and operationalised regulatory governance conditions across the policy cycle.

#### High industry involvement

The literature review identified the roles of actors, ideas, political contexts and evidence that shaped agenda setting, design and implementation of the policy cases included in this study^(13)^. In this analysis, we included policies that were already adopted thus our interest was less on the contextual factors that led to policy adoption and more on the factors that shaped how the policies were eventually designed. A key factor in the literature was the powerful role of the industry in shaping the design and implementation of policies in ways that reflect their interests and often in conflict with public health interests^(13,26,27)^. Based on this, we predict that the extent of high industry involvement may be one of the conditions that may influence food policy efficacy.

#### Strictness of regulatory design

The review highlighted multiple regulatory designs ranging from strict government command and control mandatory policies to less strict quasi-regulation in public–private partnerships and pure industry self-regulation. Critique exists for each type of regulatory design. For example, industry self-regulation is criticised for the lack of transparency and being profit centric at the expense of public interests^(28)^, while the legitimacy of public–private partnerships formed under quasi regulation is often challenged by competing interests and lack of trust and accountability between public and private stakeholders^(29)^. Similarly, mandatory policies that are underpinned by legislation, whereby the governments make, monitor and enforce the rules are critiqued for lack of flexibility and limited capacity to adequately monitor and enforce these policies^(24,30)^. We selected strictness of regulatory design as our second condition that would contribute to policy outcomes.

#### Best practice instrument design

The literature review on the regulatory governance of instrument design revealed various approaches to designing food policies, including the use of best practices^(13)^. The factors included for instrument design were the extent to which the policy was designed following best practices based on the recommendations by international organisations such as the WHO^(25)^, International Network for Food and Obesity/Non-communicable Disease Research, Monitoring, and Action Support (INFORMAS)^(29)^ and the World Cancer Research Fund International Nourishing framework^(30)^. Instrument design is crucial for policy implementation because it sets out the rules and substantive content of regulatory policies that guide the monitoring and enforcement processes^(31)^. We predict that the extent to which policies are designed following international best practices may influence policy efficacy, thus we selected best practice instrument design as the third condition.

#### Comprehensive monitoring and enforcement

The literature review identified various shortcomings and best practices in policy implementation. Based on the regulatory scaffolding framework for ensuring accountability in policies^(14)^, important factors identified were administration, monitoring and enforcement of policies. Other accountability frameworks also emphasise the crucial role of monitoring and enforcement in effecting nutrition policies^(4,32)^. We therefore selected comprehensive monitoring and comprehensive enforcement as two conditions of policy implementation that could shape policy outcomes. Comprehensive monitoring entails an independent transparent monitoring system that includes baseline data before the policy was implemented, as well as a set of measurable, time-bound process and outcome indicators^(14,17)^. Comprehensive enforcement entails availability of a complaints handling systems that has incentives and sanctions enforcement systems overseen by an independent body^(14,17)^. These practices were identified through the literature review^(22)^ and supported by the policy implementation frameworks from international organisations such as the WHO, INFORMAS and World Cancer Research Fund International nourishing framework^(4,30,32)^.

### Policy outcomes

We included three types of policy outcomes: (1) improvements in the food environment, which entails reductions in the availability, affordability or accessibility of unhealthy foods (or increases in heathy foods) after the policy was implemented, (2) improvements in behaviours, which relates to changes in consumer behaviour after the policy was implemented, such as increased purchasing of healthy foods or reduction in the consumption of unhealthy foods and (3) improvements in health outcomes, which entail improvements in nutrition-related health outcomes after the policy was implemented, such as a reduction in diet-related chronic disease mortality rates or reduction in the prevalence of obesity.

### Step 3: Calibrating conditions and outcomes

The next step of the QCA analysis involved assigning each policy case a score ranging from 0 to 1 based on the extent to which they display the conditions of interest and outcome (belong to a set of cases that show certain characteristics of condition). This process is called calibration. Scores can either be assigned in a binary manner (0 or 1) to form crisp sets or in a way where conditions are calibrated continuously between 0 and 1^(20)^, which is called fuzzy sets. In this study, we use fuzzy sets as they allow for more detail in the data^(33)^. A score of 0 indicates that the policy case does not display the condition of interest (not a member of that set), 0·33 indicates that the case displays the character of interest to a small extent (more out of the set than in), 0·66 indicates that the case displays most of the (more in the set than out and 1 indicates that the case displays all the characteristics of the set (fully in the set). Below we explain how each condition was calibrated (Supplemental file 2 presents details of how each policy case was calibrated for each of the five conditions).

#### High industry involvement

High industry involvement was calibrated as 1 if industry led the entire policy process, i.e. self-regulation; 0·66 if industry was involved in the agenda setting and design of government policy through, e.g. sitting on working groups for target setting. High industry involvement was calibrated as 0·33 if the industry was engaged as an external stakeholder that may have led to changes in some aspects of the design or implementation. Lastly, high industry involvement was calibrated as 0 in policies where the industry was consulted and involved only as an external implementer.

#### Strictness of regulatory design

We calibrated the condition of strictness of regulatory design based on three indicators: (1) the policy is underpinned by legislation; (2) the policy is mandatory and (3) rule making, monitoring and enforcement are done by government. Presence of all three indicators was calibrated as 1 as observed in mandatory policies in which governments make, monitor and enforce policies. Presence of two indicators was calibrated as 0·66 for co-regulation policies that are mandatory and underpinned by legislation, but aspects of implementation are done by industry or regulatory agencies. Presence of one indicator was calibrated as 0·33, which was observed in quasi-regulation policies such as voluntary public partnerships that involve government but are voluntary and not underpinned by legislation. Regulatory design was calibrated as 0 for policies that did not have any of the indicators such as pure self-regulation by the industry.

#### Best practice instrument design

Policy goals, standards, objectives and terms are articulated differently within the different policy domains of reformulation, labelling, marketing and taxation. For this reason, four indicators of best practice instrument design were selected for each policy domain (see Supplemental file 2). Presence of three or more best practice indicators was calibrated as 1; presence of 2 best practices indicators as 0·66 and the presence of 1 best practice indicator was calibrated as 0·33; while policy cases that did not have any of the best practice indicators were scored 0.

#### Comprehensive monitoring

We calibrated the condition of comprehensive monitoring based on three indicators: (1) existence of a monitoring system; (2) sound methods (e.g. comprehensive, transparent and independent monitoring system that includes baseline data prior to the policy as well as a set of measurable, time-bound process and outcome indicators) and (3) government or third-party (non-industry) monitoring. Presence of all three indicators was scored 1; presence of two indicators was scored 0·66 and presence of one indicator was scored 0·33. Policy cases with none of the indicators were calibrated as 0.

#### Comprehensive enforcement

We calibrated the condition of comprehensive enforcement based on three indicators: (1) existence of an enforcement system, (2) existence of sanctions and (3) government or third party (non-industry) enforcement. Presence of all three indicators was calibrated as 1; presence of two calibrated as 0·66, presence of one indicator was calibrated as 0·33 and policy cases that had none of the indicators were calibrated as 0.

#### Policy outcomes

Policy outcomes were calibrated in a binary manner. In our study, 1 relates to improvements in the food environment, behavioural outcomes or health measures detected after policy implementation. No improvements in the food environment, behavioural outcomes or health outcomes detected after policy implementation were calibrated as 0.

The literature on food policies indicates varying impacts on different outcomes. For example, changes to the food environment are more immediately observed, while changes in behaviour and health take longer^(6,7)^. In our review of the literature, more articles reported the impacts of food policies on the food environment compared with behaviour and health. Some policy cases had mixed results, for example, one study indicating a positive impact on the food environment, and another analysis of the same policy case indicating a failure to demonstrate positive impacts on behavioural outcomes. In these instances, we calibrated the policy cases using the most robust measure based on literature. For example, a nutrition labelling policy that had a positive outcome on the ‘number of labels seen by customers’ but had no effect on ‘energies purchased’ was coded as 0 because energies purchased is a more robust measure of impact of a nutrition label^(34)^. A summary of calibration of policy outcomes and data sources is shown in Supplemental file 3.

### Step 4: Analysing of conditions

Assigning each policy case a score ranging from 0 to 1 produces a data table matrix (see Supplemental File 4). The data table is then uploaded into fsQCA software to analyse for necessary and sufficient conditions. Standard QCA analysis entails analysing the data to identify the necessary and sufficient regulatory governance conditions that are present when there are positive policy outcomes, and when the outcomes do not occur, i.e. the policy does not work.

The analysis of necessary conditions identifies the conditions that must be present for the outcome to occur. For a condition to be necessary, the consistency score must be equal to or > 0·9, and the coverage must be > 0·51^(20)^. Consistency refers to the percentage of similar combinations of conditions that also display the outcome – the higher the consistency, the stronger the relationship between a necessary condition and an outcome. For necessity, coverage refers to the number of cases with the outcome that also have the condition.

The analysis of sufficient conditions identifies combinations of conditions that cause the outcome to occur. The fsQCA program produces a ‘truth table’ that shows all the ways conditions can combine to produce an outcome, regardless of whether they are observed in the data. While the truth table shows all possible configurations of conditions, further analysis focuses on causal configurations that are observed at least once in the data and those that explain a large proportion of the policy cases^(28)^. In fuzzy sets, the general rule is that a configuration must explain 75–80 % of cases to be regarded as relevant. This is called a consistency and refers to the proportion of cases in a configuration that also display the outcome^(28)^. For this analysis, the consistency threshold was set at 0·80.

## Results

The results of the QCA analysis for food policy outcomes are summarised in Table 1 and 2. Of the five regulatory governance conditions included in the analysis, only comprehensive monitoring was identified as being necessary for the policy outcome to occur (Table 1). The consistency score for monitoring was 0·94, which suggests that at least 94 % of the policy cases that were implemented using comprehensive monitoring displayed the policy outcome. The coverage was 0·75, indicating that in 75 % of the cases that had the outcome there was comprehensive monitoring.

**Table 1:** Necessary regulatory governance conditions for effective food policy

Regulatory governance conditions	Coverage	Consistency
High Industry Involvement	0·45	0·29
Strict Regulatory Design	0·82	0·72
Best practice instrument design	0·84	0·82
Comprehensive Monitoring	0·80	0·94*
Comprehensive Enforcement	0·83	0·58

* Necessary condition.

**Table 2:** Sufficient conditions for effective policies

Configurations of sufficient conditions for the policy outcome to occur	Raw coverage	Consistency
High Industry Involvement† Strict Regulatory Design* Best practice Instrument Design* Comprehensive Monitoring* Comprehensive Enforcement* Danish trans-fat ban South African Sodium Reformulation Legislation, Philadelphia Sugar Sweetened Beverage Tax, Chile labelling and Advertising Law, King Country Calorie Labelling, New York City Trans-fat Ban, Berkeley SSB Tax, United Kingdom Soft Drinks Industry Levy South Korean Food Marketing Policy Quebec Consumer Protection Act Ireland rules on food marketing to children	0·47	0·96
High Industry Involvement† Strict Regulatory Design† Best Practice Instrument Design* Comprehensive Monitoring* Comprehensive Enforcement† United Kingdom Sodium Reformulation the Food Standards Agency United States National Salt Reduction Initiative	0·17	0·82
Configurations of sufficient conditions for the policy outcome to not occur		
High Industry Involvement† Strict Regulatory Design* Best Practice Instrument Design† Comprehensive Monitoring† Comprehensive Enforcement* Quebec Consumer Protection Act Swedish Food Marketing Regulations	0·15	1
High Industry Involvement* Strict Regulatory Design† Best Practice Instrument Design† Comprehensive Monitoring† Comprehensive Enforcement† Australia Food Marketing Pledges Canadian Children’s Advertising Initiative New Zealand Broadcasting Code United States Children’s Food and Beverage Advertising Initiative Australian Food and Health Dialogue	0·48	0·82

* Presence of condition.

† Absence of condition.

Table 2 shows the configurations of conditions that were sufficient for the policy outcome to occur, and configurations of conditions that were sufficient for the policy outcome not to occur.

There were two configurations that explained a significant proportion of the policy cases and were therefore considered sufficient for the policy outcome to occur. The first configuration involved strict regulatory design, plus best practice instrument design, plus comprehensive monitoring, plus comprehensive enforcement and no high industry involvement. This configuration had a consistency score of 0·96, which means that there were improvements in the policy outcomes in 96 % of the cases that had this combination of conditions.

The second configuration of regulatory governance conditions that was sufficient for a positive policy outcome was the absence of high industry involvement, plus absence of strict regulatory design, plus the presence of best practice instrument design, plus presence of comprehensive monitoring and absence of enforcement. The consistency score for the second configuration was 0·82, and the coverage was 0·17.

While both configurations of conditions are sufficient for the outcome to occur, the first configuration is observed in more policy cases with improvements in policy outcomes and therefore more empirically robust.

There were also two configurations of conditions that were sufficient for improvements in policy outcomes not to occur (see Table 2). Hundred percent of the policy cases with absence of high industry involvement combined with strict regulatory design; absence of best practice design; absence of comprehensive monitoring and presence of comprehensive enforcement led to no improvements in the policy outcomes (consistency score = 1). The coverage for this configuration of conditions was 0·15. These findings suggest that policies without comprehensive monitoring or best practice design do not produce improvements in outcomes even in the presence of a strict regulatory design and comprehensive enforcement.

The second configuration of conditions that led to the outcome not to occur had high industry involvement combined with the absence of strict regulatory design, absence of best practice design, absence of comprehensive enforcement and absence of comprehensive enforcement. About 82 % of the policy cases that did not display the population nutrition outcomes had this configuration. This configuration also has higher coverage than the first configuration (0·48) which shows that it occurs in more cases and therefore more empirically robust than the first configuration.

## Discussion

This study used QCA to identify regulatory governance conditions across the policy cycle that lead to improvements in food policy outcomes (food environment, consumer behaviour and health). Using a theory-informed analytical framework, we extracted regulatory governance conditions from peer reviewed and grey literature on implemented food marketing, nutrition labelling and food reformulation and taxation policies. The conditions identified for analysis were high industry involvement, strict regulatory design, best practice instrument design, comprehensive monitoring and comprehensive enforcement. We identified comprehensive monitoring a necessary condition for positive policy outcomes to occur. We identified two combinations of conditions that are always present when positive policy outcomes are also present and two combinations of conditions that are always present when the outcome is absent.

With regard to necessity, of the five regulatory governance conditions we examined, comprehensive monitoring was the only condition that must be present for a policy to have a positive impact. These findings suggest that whenever there is comprehensive monitoring, food policies work for nutrition outcomes. Comprehensive monitoring provides mechanisms to assess and report on industry compliance with policy interventions and was considered present when there was a monitoring system in place that was overseen by a body independent of industry and was based on sound processes (e.g. evidence-based baseline measures, public reporting). While QCA does not elucidate why a specific condition was necessary, we know that comprehensive monitoring is considered a policy implementation best practice and has been emphasised as important in various frameworks for improving efficacy of policies^(14,17,32,35)^. This study helps to strengthen these recommendations by providing empirical evidence of a causal relationship between comprehensive monitoring and positive policy outcomes. Future research exploring the mechanisms by which monitoring enhances policy efficacy would be valuable.

While our analysis finds that comprehensive monitoring is a necessary condition for food policies to have positive outcomes, the presence of comprehensive monitoring alone does not cause food policy outcomes to occur unless combined with other regulatory governance conditions. Our findings suggest that as long as there is absence of high industry involvement, positive policy outcomes were observed in policy cases that have a combination of comprehensive monitoring and are designed using best practices display outcomes even in the absence of a strict regulatory design and enforcement. This pattern was observed in government led sodium reformulation initiatives such as the United States National Sodium Reduction initiative where even though there was no legislation in place, the development and monitoring of targets were not entirely in the hands of the food industry^(36–38)^. In these voluntary sodium reduction policies, government and regulatory agencies such as the Food Standards Agency steer the policy process by setting targets and monitoring the policies. These findings suggest that food policies can work even in the absence of legislation as long as critical parts of the policy process such deciding on instrument design and monitoring are government led. Setting up legislation can be an onerous and expensive process, and often governments are not able to monitor the performance of policies which renders them ineffective. The findings of this study propose that government involvement can have a positive effect on the policy process without requiring mandatory legislation. The findings are supported by the theories of smart^(39)^ and responsive regulation^(24)^ which posit involvement of multiple regulatory actors and use of non-legislative mechanisms to influence industry behaviour as an alternative to mandatory government regulation in some situations.

However, while policies can work without legislation, our findings suggest that much more often, successful policies do involve legislation combined with absence of high industry involvement plus strict regulatory design, best practice instrument design and comprehensive monitoring and enforcement. In most public–private partnerships, the industry has more power and resources to influence the design and implementation of policies compared with governments. Consequently, these types of policies have been shown to have poor design and lack monitoring and enforcement mechanisms to hold the industry accountable^(40–42)^ Therefore, the second combination of conditions that is often observed in government-led policies that are underpinned by legislation such as taxation policies^(43)^, statutory food marketing^(44)^ and trans-fat bans^(45)^ is still a better pathway to successful policies. These findings provide empirical support for food and nutrition policy recommendations made by international health organisations and the international literature^(3,46)^. For example, the WHO recommends that food policy must be led by government and industry efforts should simply complement or support government efforts^(25)^.

Mandatory government-led legislation is also present in one of the configurations that cause the positive policy outcome not to occur. This was observed in government policies that did not use best practice design and comprehensive monitoring, such as the Swedish food marketing regulations^(47)^ and the Quebec Consumer Protection Act^(17)^. This indicates just how important the presence of comprehensive monitoring and best practice instrument design are for policies to work, as policies can still fail even in there are mandatory government policies that are underpinned by legislation.

Overall, the findings from this study support the hypothesis that the regulatory governance of the policy cycle from agenda setting through to formulation and implementation shapes policy outcomes. While our study has indicated that regulatory governance has an effect on policy outcomes, it is important to note that there may be other factors that may influence policy outcomes including the type of policy, existence of other complementary policies and the nature of the policy domain itself. For example, empirical work by Hyseni et al found that taxes and mandatory reformulation policies were more effective than information-type policies such as labelling^(6,48)^. Similarly, policies that are implemented as part of broader strategies with complementing policies for example a sugar tax that is also accompanied by a labelling policy and information campaigns^(49)^.

### Implications for policy

This study has four key implications for policy. The first relates to high industry involvement in the policy process. Our findings provide empirical evidence that food policies work better for population nutrition outcomes when the food industry is not involved at the design stage of the policy. Government-led policy development is much more effective.

Second, we see that positive policy outcomes do not occur without comprehensive monitoring. This highlights a need to pay attention to the monitoring of food policies. This is applicable to both future policy development and current policies. Future policies must be implemented with comprehensive monitoring plans as defined by international standards and existing literature which can include monitoring that is independent of industry and sound processes (e.g. evidence-based baseline measures, public reporting), frequent and transparent reviews^(15–17)^. For existing policies that have shortcomings in the monitoring process, these findings are an indication that improving monitoring is a good first step towards improving the efficacy of policies. This approach to improving the performance of voluntary policies is also supported by the responsive regulation literature, which suggests the use of third parties such as regulatory agencies and civil society in monitoring and enforcement of policies^(24)^.

Third, this study suggests that with best practice instrument design and comprehensive monitoring, positive policy outcomes can be achieved even in the absence of strict regulatory designs such as legislation. Therefore, the efficacy of existing voluntary policies can be improved by strengthening instrument design, for example setting evidence-based targets and using stringent standards to monitor performance.

Finally, there is an alternate pathway to effective policy, but seldom seen. In instances where it is difficult to implement legislation, best practice instrument design paired with comprehensive monitoring provides the best chance of getting effective policy especially when high industry involvement is minimal.

### Limitations and implications for further research

While this study makes novel contributions on why certain policies work and others fail, there were some limitations. QCA is an explorative approach therefore the identified causal combinations should be examined further using other methods such as in-depth case studies. As this analysis was part of a bigger project, our next step is to combine the QCA findings with in-depth case studies in a specific jurisdiction. There is a limitation in the number of conditions that can be included in QCA models; therefore, there are factors that may shape policy efficacy that have not been included in this model such as the contextual factors during the agenda setting stage of the policy process, type of policy and population groups. Despite this limitation, this study gives valuable insights on the relationship between policy formulation, design and implementation processes on population nutrition outcomes. We used secondary data to conceptualise the conditions and policy outcomes. This introduces publication and reporting bias, consequently were not able to differentiate between policies that are highly effective and policies that are not effective. In addition, it must be acknowledged that the construction of conditions has a degree of researcher subjectivity. To mitigate these limitations, we sought additional information from policy documents whenever it was possible, and we kept the data calibration process transparent, theory-informed and all decisions have been recorded and submitted as supplementary material. Finally, existing literature on food policies that we accessed did not always have complete information on policy implementation. We excluded many policy cases due to the lack of complete information, and while the sample size meets the medium sample size requirement for conducting QCA, having more policy cases may have enabled identification of more causal combinations. This highlights a need for more research on the regulatory governance factors that shape the design and implementation of food policies and how these relate to policy outcomes.

## Conclusion

This study establishes causal links between regulatory governance conditions and population nutrition outcomes from a multicausality perspective. We identify key ingredients for food policies to work effectively and make recommendations for current policies. This paper also explores and illustrates the utility of QCA for public health nutrition research, demonstrating positive advances in knowledge generation and understanding of food policy efficacy.

## Supplementary material

For supplementary material accompanying this paper visit https://doi.org/10.1017/S1368980021004730. click here to view supplementary material
