# Supplementary material for: The regulatory governance conditions that lead to food policies achieving improvements in population nutrition outcomes: a qualitative comparative analysis
Source: Public Health Nutr. 2021 Dec 7;25(5):1395–405. doi: 10.1017/S1368980021004730 (PMC9991667; doi:10.1017/S1368980021004730)
Supplement: Supplementary file 1 [file S1368980021004730sup.zip › S1368980021004730sup001.docx]

*Supplementary file 2: Indicators with calibration score for each policy*

| Policy case | Industry engagement | Strict regulatory design | Best practice instrument design^a^ | Comprehensive monitoring | Comprehensive enforcement |
| --- | --- | --- | --- | --- | --- |
| Australian Health Star rating | Calibrate as .66  Industry involved in governance of the initiative and at implementation | Calibrate as .33  One out of three indicators for the involvement of government in quasi-regulation | Calibrate as .33 Nutrient criteria developed by an independent body But there are challenges with design – it requires interpretation, monochrome and food companies can choose design. | Calibrate as 1  Existence of a monitoring system  independent monitoring done by an NGO  Public and frequent reports on the performance of the scheme | Calibrate as .33  There is a complaints handling mechanism but no sanctions except listed on HSR website |
| Australian Food Marketing codes | Calibrated as 1  Industry-led policy | calibrate as 0  None of the indicators – the policy is pure self-regulation | Calibrate as 0  No indicators for design- no independent criteria , no aim to reduce exposure,  Only for children under 12. | Calibrate as .33  One indicator for comprehensive monitoring - existence of monitoring system but its complaints based, no independent body , no baseline | Calibrate as 0  not enforced |
| Australian Food & health dialogue | Calibrate as .66  Industry involved in governance & at implementation | Calibrate as .33  One out of three indicators for the involvement of government in quasi-regulation | Calibrate as .33  One indicator of best practice design - having targets. | Calibrate as .33  Monitoring mentioned but not specific  No baseline data  No independent third party | Calibrate as 0  not enforced |
| Berkeley SSB tax | Calibrate as 0  Industry involved at implementation, no reported impact on design or implementation. | Calibrate as 1  All three indicators are present - Mandatory policy, led by government policy  Underpinned by legislation | Calibrate as .66  Two indicators of best practice design, Excises tax, earmarked, content based, does not result in 20% increase | Calibrate as 1  Three indicators of comprehensive monitoring. There is a monitoring system, Annual verification, independent stakeholder by city manager, independent from industry | Calibrate as 1  Three indicators present. Existence of an enforcement system, independent enforcement body and availability of sanctions. |
| Brazil sodium reformulation | Calibrate as .66  Industry involved in governance of the public private partnership with the ministry of health | Calibrate as .33  One out of three indicators for the involvement of government in quasi-regulation | Calibrate as 1  Three indicators of good practice design  Population intake goal  Time-based targets  Evidence-based baseline measures | Calibrate as 1  Three indicators  Existence of a monitoring system, Independent  3^rd^ party/government monitoring  Baseline data | Calibrate as 0  not enforced |
| Canada sodium reformulation | Calibrate as .66  Industry involved in governance as they constituted about one-quarter of the Health Canada Sodium working group. | Calibrate as .33  One out of three indicators for the involvement of government in quasi-regulation | Calibrate as 1  Three indicators of good practice design  Population intake goal  Time-based targets  Evidence-based baseline measures | Calibrate as 1  Three indicators  Existence of a monitoring system, Independent  3rd party/government monitoring  Baseline data | Calibrate as 0  not enforced |
| Canadian Food marketing codes | Calibrated as 1  Industry-led policy | calibrate as 0  None of the indicators – the policy is pure self-regulation | Calibrate as 0  Industry nutrient criteria, do not aim to reduce exposure, goals Industry criteria  Doesn’t cover all forms of marketing | Calibrate as .33  One indicator for existence of monitoring system but its complaints based, no independent body , no baseline | Calibrate as 0  not enforced |
| Chile Food Labelling and Advertising law | Calibrate as 0  Industry involved as an external stakeholder in implementation, no reported changes to the policy. | Calibrate as 1  All three indicators are present - Mandatory policy, led by government policy  Underpinned by legislation | Calibrate as 1  Three indicators of best practice design Independent nutrient criteria, label design, non-interpretive, covers various platforms | Calibrate as 1 Existence of a monitoring system  monitoring system, government | Calibrate as 1  Three indicators present. Existence of an enforcement system, independent enforcement body and availability of sanctions. |
| Danish wholegrain logo | Calibrate as .66  Industry involved in governance & at implementation | Calibrate as .33  One out of three indicators for the involvement of government in quasi-regulation | Calibrate as 1  Three best practice design indicators  Logo type on healthy food, doesn’t require interpretation, government nutrient criteria. | Calibrate as .66  Two indicators  There is a Monitoring system, multiple methods are used, not independent of industry | Calibrate as 0  not enforced |
| Danish trans-fat ban | Calibrate as .33  Industry involved as an external stakeholder before implementation. | Calibrate as 1  All three indicators are present - Mandatory policy, led by government policy  Underpinned by legislation | Calibrate as 1  Three indicators of best practice design - Maximum limits, Applies to all food stuffs  Baseline measures | Calibrate as 1  Three indicators present.  Existence of a monitoring system, independent monitoring  Consistent measuring of population trans-fat intake | Calibrate as 1  Three indicators present. Existence of an enforcement system, independent enforcement body and availability of sanctions. |
| Dutch choices logo | Calibrate as .66  Industry policy that was later approved by government | Calibrate as .33  One out of three indicators for the involvement of government in quasi-regulation | Calibrate as 1  Three best practices design indicators present  Logo type on healthy food, doesn’t require interpretation, WHO criteria | Calibrate as 1  Three indicators for comprehensive monitoring  Existence of a monitoring system,  Monitored by an independent scientific committee & reviewed every four years | Calibrate as 0  not enforced |
| The Broadcasting Authority of Ireland (BAI) code | Calibrate as 0  Industry involved at implementation, no reported impact on design or implementation. | calibrate as 1  All three indicators are present - Mandatory policy, led by government policy  Underpinned by legislation | Calibrate as .1  Goal to reduce exposure , applies to children under 18, independent nutrient criteria - | Calibrate as 1  existence of a monitoring system, done by an external body or government | Calibrate as 1  Three indicators of comprehensive enforcement - there is an enforcement system, independent from the industry and there are sanctions for noncompliance. |
| King County Calorie Labelling | Calibrate as 0  Industry involved at implementation, no reported impact on design or implementation. | Calibrate as 1  All three indicators are present - Mandatory policy, led by government policy  Underpinned by legislation | Calibrate as .66 specifies the outlets the regulations apply to, includes drive through, and specifies the nutrients, criteria up to the establishment | Calibrate as 1  Existence of a monitoring system, done by an external body or government | Calibrate as .66  Two indicators  Enforced by department of health but no specific sanctions/fines |
| New South Wales calorie labelling | Calibrate as 0  Industry involved at implementation, no reported impact on design or implementation. | calibrate as 1  All three indicators are present - Mandatory policy, led by government policy  Underpinned by legislation | Calibrate as .66  Two indicators  Specifies the outlets affected, challenges with legibility and display. | Calibrate as 1  existence of a monitoring system, done by an external body or government | Calibrate as .66  Two indicators of comprehensive enforcement - there is an enforcement system but it’ s weak (written notice and assistance with implementation) |
| New York City Trans-fat Ban | Calibrate as .33  Industry involved as an external stakeholder prior to implementation. Timelines were adjusted based on these consultations. | Calibrate as 1  All three indicators are present - Mandatory policy, led by government policy  Underpinned by legislation | Calibrate as .66  Two indicators for having upper limits and articulated policy goals but only applies to restaurants and does not include packaged food | Calibrate as 1  Three indicators there is an existence of a monitoring system, done by an external body or government, measured against baseline | Calibrate as 1  Three indicators present. Existence of an enforcement system, independent enforcement body and availability of sanctions. |
| New York City Calorie labelling | Calibrate as .33  Industry involved as an external stakeholder in implementation. Policy amended after a court ruling. | Calibrate as 1  All three indicators are present - Mandatory policy, led by government policy  Underpinned by legislation | Calibrate as 1. Three indicators for best practice design - Prominent,  Readable, expression of daily intakes | Calibrate as 1  Three indicators Independent Monitoring system  Monitoring by DOH | Calibrate as 1  Three indicators of comprehensive enforcement - there is an enforcement system, independent from the industry and there are sanctions for noncompliance. |
| New Zealand food marketing self-regulation | Calibrate as 1  Industry-led policy | Calibrate as 0  None of the indicators – the policy is pure self-regulation | Calibrate as .33  One indicator of design best practices -nutrient criteria, no goals to reduce exposure,  Doesn’t cover all media | Calibrate as .33  One indicator for existence of monitoring system but its complaints based, no independent body , no baseline | Calibrate as 0  not enforced |
| Philadelphia SSB tax | Calibrate as 0  Industry involved as an external stakeholder in implementation. There was a court case but it did not lead to changes in the design of the policy. | Calibrate as 1  All three indicators are present - Mandatory policy, led by government policy  Underpinned by legislation | Calibrate as 1  Three indicators of good tax design  Excise tax, if passed through it will result in a 20% increase, earmarked | Calibrate as 1  Three indicators of good monitoring  Monitored through tax returns, there is a system, independent from industry | Calibrate as 1  Three indicators present. Existence of an enforcement system, independent enforcement body and availability of sanctions. |
| Quebec | Calibrate as 0  Industry involved at implementation, no reported impact on design or implementation. | Calibrate as 1  All three indicators are present - Mandatory policy, led by government policy  Underpinned by legislation | Calibrate as .33  One indicator of best practice design  Seeks to protect from all exposure commercial marketing, but excludes some platforms, only shows that target children under 13. | Calibrate as .33  There is a monitoring system but it is complaints based, no routine measurement of the impact of food advertising on children. | Calibrate as .66  Two indicators present  There is an enforcement system  Independent from industry  There is a range of enforcement options but they are not used |
| South Africa sodium reformulation | Calibrate as .33  Industry engaged before implementation which led to changes in some of the targets. | Calibrate as 1  Three indicators are present  Mandatory policy, led by government policy  Underpinned by legislation | Calibrate as 1  Three indicators of good practice design  Population intake goal  Time-based targets  Evidence-based baseline measures | Calibrate as 1  Existence of a monitoring system, Independent monitoring  Plan to measure population salt intake and content analyses | Calibrate as 1  Three indicators present. Existence of an enforcement system, independent enforcement body and availability of sanctions. |
| South Korean Special Act on Safety Management of Children’s Dietary Life (Food advertising) | Calibrate as 0  Industry involved at implementation, no reported impact on design or implementation. | calibrate as 1  All three indicators are present - Mandatory policy, led by government policy  Underpinned by legislation | Calibrate as .66  Two indicators of best practice design – there is goal to reduce exposure to unhealthy food advertising, Independent nutrient criteria but doesn’t cover all platforms. | Calibrate as 1  existence of a monitoring system, done by an external body or government | Calibrate as 1  Three indicators of comprehensive enforcement - there is an enforcement system, independent from the industry and there are sanctions for noncompliance. |
| Spanish voluntary codes for food marketing | Calibrate as .66  Aspects of the policy are sanctioned by the ministry of health | calibrate as 0  None of the indicators – the policy is pure self-regulation | Calibrate as 0  No goal to reduce exposure, limited to 15 years, no nutrient standards for what should be advertised, does not cover all forms of marketing | Calibrate as 1  Three indicators for comprehensive monitoring present. There is an apriori system where ads are screened before advertisements – there is a monitoring commission | Calibrate as .66  There is an enforcement system and fines but these only apply to organizations that are part of the pledge. |
| Sweden food marketing regulations | Calibrate as 0  Industry involved at implementation, no reported impact on design or implementation. | Calibrate as 1  All three indicators are present - Mandatory policy, led by government policy  Underpinned by legislation | Calibrate as .33  Only one indicator - aims to reduce exposure to all marketing but only goes till the age of 12, broadcasting laws don’t apply to non-broadcast media | Calibrate as 0  No indicator of comprehensive monitoring- No systematic monitoring related to unhealthy food or exposure to food marketing | Calibrate as .66  Two out of three indicators present  There is an enforcement system  Independent from industry  There is a range of enforcement options but they are not used |
| United Kingdom food marketing | Calibrate as 0  Industry involved as an external stakeholder in implementation. | calibrate as 1  All three indicators are present - Mandatory policy, led by government policy  Underpinned by legislation | Calibrate as 1  Goal to reduce exposure, includes multiple platforms, independent nutrient criteria to evaluate foods that can be marketed | Calibrate as .66  Two indicators present There is systematic monitoring  Done by an independent body  No impact assessments of food advertising | Calibrate as 1  Three indicators present. Existence of an enforcement system, independent enforcement body and availability of sanctions. |
| United Kingdom Soft drinks levy | Calibrate as 0  Industry involved at implementation, no reported impact on design or implementation. | Calibrate as 1  All three indicators are present - Mandatory policy, led by government policy  Underpinned by legislation | Calibrate as .66  Two-Tiered based on content, levied on manufacturer, not an excise tax | Calibrate as 1  Three indicators present  Existence of a monitoring system, Independent  3rd party/government | Calibrate as 1  Three out of three indicators present. Existence of an enforcement system, independent enforcement body and availability of sanctions. |
| United Kingdom sodium reformulation (Food Standards Agency) | Calibrate as .33  Led by an independent Food Standards Agency with industry consulted as an external stakeholder. | Calibrate as .33  One out of three indicators for the involvement of government in quasi-regulation | Calibrate as 1  Three indicators of good practice design  Population intake goal  Time-based targets  Population intake goal | Calibrate as 1  Three indicators present  Existence of a monitoring system, Independent  3rd party/government  24-hr urine samples to measure population intake | Calibrate as 0  not enforced |
| United Kingdom sodium reformulation (public health responsibility deal) | Calibrate as .66  Industry involved in the governance of the partnership. | Calibrate as .33  One out of three indicators for the involvement of government in quasi-regulation | Calibrate as .66  One indicator of best practice design. Targets not consistent and reported to be low. | Calibrate as .33  One indicator for existence of a monitoring system. It is not independent. Self-reports by the industry, not consistent | Calibrate as 0  not enforced |
| United States National Sodium Initiative | Calibrate as .33  Led by the New York City Department of health and mental Hygiene with other state governments and local authorities. Industry was consulted as an external stakeholder. | calibrate as .33  One out of three indicators for the involvement of government in quasi-regulation | Calibrate as 1  Three indicators of good practice design  Population intake goal  Time-based targets  Population intake goal | Calibrate as 1  Three indicators there is an existence of a monitoring system, done by an external body or government, | Calibrate as 0  not enforced |
| United States Children’s Food and Beverage Advertising Initiative | Calibrated as 1  Industry-led policy | calibrate as 0  None of the indicators – the policy is pure self-regulation | Calibrate as 0  Industry nutrient criteria, do not aim to reduce exposure, goals Industry criteria  Doesn’t cover all forms of marketing | Calibrate as .33  One indicator for existence of monitoring system but its complaints based, no independent body , no baseline | Calibrate as 0  not enforced |

**^a^Best practices**

| **Sodium reformulation** | **Front of pack labels** | **Menu labels** | **Food marketing** | **Taxes** |
| --- | --- | --- | --- | --- |
| Time based targets for reduction | Independent nutrient criteria | Specifies they type of outlets the policy applies to | Goal to reduce exposure | Excises tax on distribution/retail |
| Baseline measures before the initiative starts | Label placed on one category of food either healthy/unhealthy | Includes energy statement | Covers all media – broadcast, non-broadcast | Must result in a 20% increase |
| Population intake goal | Label stands out | Specifies font and location | Independent nutrient criteria | Earmarked for health |
| Independent third party involved in target setting | Doesn’t require interpretation | Specifies calories per portion not 100g | Definition of children | Based on content |
