# Supplementary material for: The regulatory governance conditions that lead to food policies achieving improvements in population nutrition outcomes: a qualitative comparative analysis
Source: Public Health Nutr. 2021 Dec 7;25(5):1395–405. doi: 10.1017/S1368980021004730 (PMC9991667; doi:10.1017/S1368980021004730)
Supplement: Supplementary file 1 [file S1368980021004730sup.zip › S1368980021004730sup002.docx]

*Supplementary File 3: Summary of policy outcomes*

| Policy case | Authors | Findings | | Calibration of outcome |
| --- | --- | --- | --- | --- |
| Australia – voluntary sodium reformulation under the food and Health dialogue | Trevena et al.,(2014) | The study evaluated the effects of the food and health dialogue on sodium content of bread, cereals and processed meat between 2010 and 2013. There was a decline in mean sodium content across all three categories, 9% reduction in bread, 25% reduction in breakfast cereals and 8% in processed meats. The proportions of products that met the targets also increased. | | Assigned a score of 1 |
|  | Sparks et al., 2018 | The study evaluated the sodium levels in processed meat in Australia between 2010 and 2017 and found that the sodium content of this category continued to decline between 2013 and 2017. | |  |
|  | Levi et al., 2018 | The study evaluated the compliance of Australian soup manufacturers to the 2014 sodium targets for soups. This study found a 6% decrease in overall soup content and an increase in the proportion of categories that met the targets. | |  |
| Australia/New Zealand Health star rating front of pack label | Jones et al., 2018 | This study investigates the uptake of the voluntary health star rating Front of Pack labelling and finds that the uptake increased by 28% between 2014 and 2017 and that products that displayed the label had higher mean health star rating compared to those who did not. | | Assigned a score of 1 |
|  | Hamlin et al., 2018 | This study investigates the effects of the Health Star rating on consumer choice in New Zealand and finds the Health star rating ineffective in influencing unprompted consumer choice. The effects of the label are small in relation to other elements of the product | |  |
|  | Mhurchu et al., 2018 | This study examines the effects of the health star rating on reformulation of products and finds reformulation of Health Star rating labelled products was higher compared to non-labelled products between 2014 and 2016. | |  |
|  | Morrison et al., 2018 | This study investigated the impacts of the health star rating on nutritional content and reformulation of children’s cereals. This study found that HSR products were nutritionally better than non- HSR products and reformulation had occurred in 100% of the HSR products | |  |
| Policy case | **Authors** | **Findings** | | **Calibration of outcome** |
| Australia food marketing | Hebden et al., 2011 | This study evaluated the impact of Quick service restaurants self-regulatory on fast food advertising to children on TV from 2009 to 2010. This study found no changes in the frequency and rate of fast food advertising. | | Assigned a score of zero |
|  | Jones et al., 2010 | This study evaluate the extent of food advertising on children’s magazines and found that most of the ads were in breach of codes for advertising to children. | |  |
|  | Jones et al., 2010 | This study examined the nature of food advertising in children’s magazines and found that a majority of the branded food ads were for non-core foods and most were not clearly identified as adverts. | |  |
|  | Kelly et al., 2007 | This study examined the nature of food advertising in Australia and evaluated the against Australian Children’s Television Standards. There was a reduction in overall food advertising, however children were still highly exposed to unhealthy food | |  |
|  | King et al., 2013 | This study evaluated the impact of the advertising pledges on food and drinks ads on television. This study found that there were no significant differences in frequency of ads between 2006 and 2011. | |  |
|  | King et al., 2012 | This study compared patterns of advertising between companies that are signatories to the advertising pledges and those that not signatories. This study foundation significant reduction in non-core food by signatories compared to non-signatories. However, the rate of non-core food advertising was not different. | |  |
|  | Roberts et al., 2012 | This study evaluated compliance of children’s TV advertisements with the Children’s services standard. This study found a significant number of breaches in the amounts of advertising, the types of foods advertises and the use of marketing techniques. | |  |
|  | Roberts et al., 2014 | This study evaluated exposure to unhealthy foods through televised food advertising and found that despite the code, children continue to be exposed to unhealthy food marketing | |  |
|  | Watson et al., 2017 | This study evaluated the efficacy of the self-regulatory initiatives implemented in 2009 on reducing the rate of unhealthy food advertising to children. There was no change in the rate of unhealthy food advertising between 2009 and 2011 despite the code | |  |
| Berkeley SSB tax | Falbe et al., 2016 | This study evaluates the impact of the tax on SSB consumption and found a 21% decrease in SSB consumption and 63% increase in water consumption | | Assigned a score of 1 |
|  | Lee et al., 2019 | This study estimated the changes in SSB and water consumption 3 years the tax. This study found a sustained decrease in SSB consumption and an increase in water consumption. | |  |
| Policy Case | **Authors** | **Findings** | **Calibration of outcomes** | |
|  | Silver et al., 2017 | This study measures the associations between the tax and beverage prices, sales, spending and beverages intake. The prices of SSBs increased but not across all store types, sales of taxed beverages declined, the sales of untaxed beverages increased but the reported intake did not increase significantly. |  | |
| Brazil voluntary sodium reformulation | Nilson et al., 2017 | This study measure sodium content in food categories from 2011 to 2017. This study found significant reduction in medium sodium content in over half of the food categories and by 2017 most of the categories had met the regional targets | | Assigned a score of 1 |
| Canada voluntary sodium reformulation | Health Canada, 2017 | This evaluation report looks at the proportion of product categories that met the targets. This evaluation found that only 30% of food product categories lowered the salt contents from maximum level and 48% did not make any progress towards the targets. | | Assigned a score of 0 |
| Canadian Children’s advertising initiative | Potvin Kent et al., 2013 | This study compared nutrient content of food TV ads between the Quebec consumer protection act and the Canadian self-regulatory policy. The Quebec advertisements were significantly higher in total fat, saturated fat, and protein per 100 g compared to the English provinces. However, Quebec had a lower proportion of ads considered high fat and high sodium and the food ads in Quebec were considered marginally healthier than the English jurisdiction. | | Assigned a score of 0 |
|  | Potvin Kent et al., 2018 | This study assessed the effectiveness of the CAI on restrict advertising on children’s websites. This study found that the ads on children’s websites were high in sodium, fat and free sugars. Overall, the study regulations did not reduce advertisement of unhealthy food on children’s websites | |  |
|  | Potvin Kent et al., 2018 | This study evaluated the impact of the self-regulation initiative on television food and beverage advertising. This study found that the regulations did not have an impact on marketing practices such as use of promotions and media characters and the nutritional quality of the advertised foods. | |  |
|  | Potvin Kent et al., 2014 | This study evaluated the volume, marketing techniques and nutritional quality of foods marketed to children on television. This study found a decrease in volume on children’s specialty channels , however there was more use of marketing techniques and no changes in nutritional quality | |  |
| Policy Case | **Authors** | **Findings** | | **Calibration of Outcomes** |
| Chilean Food Labelling and Advertising Law | Reyes et al., 2020 | This study evaluated the impact of the law on the nutrient content of packaged food.  This study found that there was a significant decrease in the amount of sugars and sodium in packaged foods | |  |
|  | Carpentier et al., 2020 | This study examined the effect of the law on exposure to unhealthy food via advertising. There was a decrease in exposure among pre-schoolers and adolescents | |  |
|  | Correa et al., 2020 | This study evaluated the nature of food advertising to children before and after the implementation of the Chilean law. This study found a decline in the prevalence of High energy, fat, salt and sugar foods. | |  |
| Danish Wholegrain partnership | Greve & Ness | This study evaluates the wholegrain partnership in Denmark and finds that the partnership had an impact in whole grain consumption, the number of whole grain products available | | Assigned a score of 1 for awareness and use of labels and calories purchased. |
| Denmark Trans-fat ban | Leth et al., 2006 | This study estimates the effects of the regulation on trans fatty acid content in Danish foods and finds that the content has been reduced significantly in products that were initially very high in trans-fats | | Assigned a score of 1 |
|  | Restepo & Rieger, 2016 | This study estimates impact of trans-fat regulations on Cardiovascular diseases mortality rates. This study found that there was a decrease in CVD mortality after the implementation of the trans-fat regulations | |  |
| Dutch choices logo | Smed et al., 2019 | This study examined the impact of the Choices’ logo on household purchase patterns  And found that there was an increase in the volume share purchased. | | Assigned a score of 1 |
| Policy Case | **Authors** | **Findings** | | **Calibration of Outcomes** |
| Irish Broadcasting Code | Tatlow-Golden et al., 2015 | This study evaluates the nature of television advertisements to children. Most of the advertisements on children’s television comply with the legislation, however children are still exposed to adverting through adult television. | | Assigned a score of 1 because Children’s shows which are targeted by the policy are compliant |
| King county calorie labelling | Bruemmer et al., 2012 | This study evaluated the nutritional content of meals at regulated restaurants 18 months after the implementation of the policy. This study found that there was a decrease in energy, saturated fat and sodium in meals that were there before the regulations. | | Assigned a score of 1 |
|  | Chen et al, 2015 | This study examined calorie information awareness and use before and after the policy. This study found that the proportion of people who saw and used calorie information increased from 8.1% to 24.8% | |  |
|  | Finkelstein et al., 2011 | This study measured the impact of the Calorie labelling on purchasing behaviour immediately after the calorie labelling law. This study found no significant changes on calories purchased. | |  |
|  | Krieger et al., 2013 | This study measured the impact of the policy eighteen months after implementation and found that calories purchased decrease 18 months after implementation and the awareness of calorie information increased. | |  |
|  | Tandon et al., 2011 | This study evaluated the impact of the regulation on compared the calories purchased by children, this study found increased awareness of labels, decrease calories purchased in adults and no differences for children | |  |
| Policy Case | **Authors** | **Findings** | | **Calibration of Outcomes** |
| New York trans-fat ban | Restepo & Rieger, 2016 | This study estimates impact of trans-fat regulations on Cardiovascular diseases mortality rates. This study found a 4% reduction in CVD mortality rates. | | Assigned a score of 1 |
|  | Sood et al., 2014 | This study examines compliance to the trans-fat ban regulations in Nassau County and finds high compliance rates to the legislation. | |  |
|  | Wright et al., 2019 | This study estimates the impacts on New York City trans-fat ban on serum trans-fatty acid levels in adults and finds high declines (up to 61, 6%) among frequent restaurant eaters. | |  |
| New York City Calorie labelling | Dumanovsky et al., 2010 | This study evaluated consumer awareness of Calorie labels after implementation and found that, there was increase in the number of people who see and use the labels. | | Contradicting outcomes excluded in the final analysis |
|  | Dumanovsky et al., 2011 | This study evaluated the nutrient energy content of lunch time meals purchased after the implementation of the calorie labelling laws. This study found no changes in mean calories purchased however there were differences across the chains, two chains showed that accounted for the majority of the survey showed a decrease while there was an increase in one. When variables such as sex and neighbourhood poverty are held constant, there is a small decline for the whole sample. In addition, customers who report to use calorie labels purchase fewer calories. | |  |
|  | Elbel et al., 2009 | This study examined the impact of calorie labels on fast food purchases and found that those who saw the labels reported that they influenced their food choices but there was no change in calories purchased | |  |
|  | Elbel et al., 2011 | This study evaluated the impact of calorie labels on fast-food calories purchased by adolescents and children. This study found no significant differences in calories purchased | |  |
| Policy Case | **Authors** | **Findings** | | **Calibration of Outcomes** |
| New South wales Calorie labelling | Wellard et al., 2015 | This study examines availability and accessibility of calorie labelling information after the implementation of the regulation. This study found that more information was available after the menu labelling implementation, however the information was incomplete. | | Contradicting outcomes excluded in the final analysis |
|  | Wellard et al., 2017 | This study evaluated the nutrient composition of fast foods after the introduction of calorie labels. This study found no significant decreases in energy content. | |  |
|  | NSW Food authority | A formal evaluation of the policy after a year after implementation found that there was a decrease in median calories purchased. | |  |
| New Zealand food marketing | Vandevijvere et al., 2017 | This study examined the extent, nature and impact of food advertising to children, This study found that children were highly exposed to unhealthy food and more than 80% of the unhealthy food advertisements were on children’s TV shows. | | Assigned a score of 0 |
| Philadelphia SSB tax | Roberto et al., 2019 | This study compared changes in beverage prices before and after the implementation of the tax. This study found that the mean price per ounce increased in all stores after the tax and the beverages volume sales declines. | | Assigned a score of 1 |
|  | Zhong et al., 2018 | This study evaluated the immediate impact of the tax on consumption of soda, energy drinks, fruit drinks and bottled water. This study found that within the first two months the odds of daily consuming regular soda were 40% lower than In other cities, energy drinks 64% lower and water 58% higher. | |  |
| Quebec Consumer Protection Act | Potvin Kent et al., 2013 | This study compared nutrient content of food TV ads between the Quebec consumer protection act and the Canadian self-regulatory policy. The Quebec advertisements were significantly higher in total fat, saturated fat, and protein per 100 g compared to the English provinces. However, Quebec had a lower proportion of ads considered high fat and high sodium and the food ads in Quebec were considered marginally healthier than the English jurisdiction. | | Assigned a score of 0 for nutrient content |
| South African Sodium reformulation legislation | Peters et al., 2017 | This study assessed early compliance to sodium reduction and found that 67% of the food products met or were below the targeted sodium levels during the early stages of the implementation. | | Assigned a score of 1 |
| Policy Case | **Authors** | **Findings** | | **Calibration of Outcomes** |
| South Korean Special Act on Safety Management of Children’s Dietary Life | Kim et al., 2012 | This study evaluated the impact of the regulations on South Korean food company practices. This study found that the advertising budgets for unhealthy food decreased while that of healthy food increased. | | Assigned a score of 1 |
|  | Lee et al., 2017 | This study evaluated the impacts of the law on the food environment. This study found that companies changed their products to meet the nutritional criteria. | |  |
| Spanish self-regulation code | Leon-Flandez et al., 2017 | This study compared the compliance of food ads directed to children in 2012 to 2008 This study found that noncompliance was higher in 2012 especially on children’s channels and outside protected slots | | Assigned a score of 0 |
|  | Ramos et al., 2015 | This study examines strategies used in in food advertising campaigns and how these breach the self-regulatory assigned a score of. This study found that ads referred to social media pages to create feedback and the food were high in sugars and saturated fats. | |  |
|  | Romero et al., 2009 | This study evaluated compliance to the Spanish self-regulatory code and found non-compliance levels high. | |  |
|  | Royo-Bordonada et al., 2016 | This study evaluated the nutritional content of food advertised on Spanish television in 2012 using different nutrient profiles. Both profiles found that the majority of the foods were non-core and high in fat, sugar and salt. | |  |
| Sweden | Sandberg et al., 2011 | This study examine the extent of food marketing to children looking at television, internet and the use of marketing techniques. This study found that the food marketed to children is unhealthy. There was also high usage of marketing techniques such as brand mascots. | | Assigned a score of 0 |
| United Kingdom sodium reformulation under the food standards agency | Eyles et al., 2013 | This study evaluated the content of sodium in processed food between 2006 and 2011 and found that there was a 7% reduction in weighted sodium content of food between 2006 and 2011. | | Assigned a score of 1 |
|  | Wyness et al., 2011 | This study evaluated the effectiveness of the FSA sodium reduction initiative and found substantial reductions in some food products. | |  |
|  | Shankar et al., 2012 | This study examined the trends in salt intake between 2003 and 2007. There was a 10% reduction in overall salt intake. | |  |
| Policy Case | **Authors** | **Findings** | | **Calibration of Outcomes** |
| United Kingdom sodium reformulation under the public health responsibility deal | Laverty et al., 2019 | This study estimates the impact of the responsibility deal on sodium intake and finds that population-level salt intake in England slowed significantly after implementation of the Public Health Responsibility Deal in 2011 | | Assigned a score of 0 |
|  | MacGregor et al., 2015 | This study observes that there was a loss of 0.9g a day loss in reduction of salt after the responsibility deal | |  |
| United States National Salt reduction initiative | Curtis et al., 2016 | The study assess the industry’s progress in reducing sodium content between 2009 and 2014, and finds that there was a 6.8% decline in weighted mean sodium density in almost half of all food categories. | | Assigned a score of 1 |
| United kingdom food marketing restrictions | Boyland et al., 2011 | This study examined the extent of food adverting on UK TV following the implementation of food marketing policy. This study found that despite the regulations children were exposed to more unhealthy food marketing | | Assigned a score of 0 for reduction in exposure |
|  | Whalen et al., 2019 | This study examines food advertising on UK TV before and after the regulation. Non-core food advertising decreased while core food adverts increased. There were still high rates of food adverts and non-core foods during children’s peak viewing times. | |  |
| United states CFBAI |  |  | |  |
|  | Harris et al., 2015 | This study evaluates child targeted on television before and after CFBAI. This study found that the companies comply with the pledges to meet minimum requirements but there are still high rates of non-nutritious foods that are being advertised. | | Assigned a score of 0 for impacts on exposure to fast food ads |
|  | Bernhard et al., 2013 | This study compared the fast food television advertisements for children to fast food television ads for adults and found that children’s ads emphasized toys and give away of products rather than the actual food, which is in conflict with the pledges. | |  |
|  | Kunkel et al., 2014 | This study examines the use of child-targeted techniques and media placements to advertise candy ion us television. This study found that although CFBAI companies complied with advertising on child targeted networks, children were still exposed to the ads placed on non-children’s channels. | |  |
|  | Kunkel et al., 2013 | This study evaluated the impact of the CFBAI on advertisements on Spanish television. This study found that the volume of advertisements was lower on Spanish television compared to English television, however the nutritional quality of the foods advertised on Spanish television was poorer than English channels, indicating lack of effectiveness in the regulations. | |  |
|  | Kunkel et al., 2015 | This study investigates the nutritional quality of food before and after the implementation of self-regulation program. This study found that there were no significant improvements in the nutritional quality of food marketed to children. | |  |
|  | Powell et al., 2010 | This study examined trends in exposure to television food advertising to children. This study found that exposure to food ads fell among younger children but increased in adolescents. Exposure to sweets ads fell while exposure to fast food ads increased | |  |
|  | Powell et al., 2010 | This study examined trends nutritional content of television food adverts to children. This study found that exposure foods high in saturated fats, sugar and sodium fell by 21.1 % while exposure too fast-foods increased | |  |
|  | Powel et al., 2013 | This study evaluated the nutritional content of foods on children’s program. This study found that, the food is predominantly unhealthy for both products under self-regulation and those that are not. | |  |
| United Kingdom Soft drinks levy | Bandy et al., 2020 | This study evaluated the impact of the tax on sales volumes, sugar content and volume of sugars sold by company and categories. This study found that the volume of sugar sales per day from soft drinks decreased by 30% and the sales-weighted mean sugar content of soft drinks declined. | | Assigned a score of 1 |
