# Supplementary material for: The regulatory governance conditions that lead to food policies achieving improvements in population nutrition outcomes: a qualitative comparative analysis
Source: Public Health Nutr. 2021 Dec 7;25(5):1395–405. doi: 10.1017/S1368980021004730 (PMC9991667; doi:10.1017/S1368980021004730)
Supplement: Supplementary file 1 [file S1368980021004730sup.zip › S1368980021004730sup003.docx]

*Supplementary File 1: Summary of policy cases and secondary data sources*

| Policy case | Policy Domain | Data sources |
| --- | --- | --- |
| Australia food & Health Dialogue | Sodium Reformulation | 1. Elliot et al., (2014): A systematic interim assessment of the Australian Government’s Food and Health Dialogue 2. Grimes et al., (2018): Trends in Dietary Sodium from Food Sources in Australian Children and Adolescents from 2007 to 2011/12 3. Jones et al., (2016): Designing a Healthy Food Partnership: lessons from the Australian Food and Health Dialogue 4. Levi et al., (2018): Evaluation of Australian soup manufacturer compliance with national sodium reduction targets 5. Magnusson et al., (2015): Food Reformulation, Responsive Regulation, and “Regulatory Scaffolding”: Strengthening Performance of Salt Reduction Programs in Australia and the United Kingdom 6. Christoforou et al., (2013): Changes in the sodium content of Australian ready meals between 2008 and 2011 7. Webster et al., (2015): Salt reduction in Australia: from advocacy to action 8. Webster et al., (2012): Drop the Salt! Assessing the impact of a public health advocacy strategy on Australian government policy on salt |
| Australian Health Star Rating | Labelling – Front of Pack | 1. Health Star Rating Advisory Committee (2017): Two year progress review report on the implementation of the Health Star Rating system – June 2014 – June 2016 2. Jones et al., (2018): Uptake of Australia’s Health Star Rating System 3. Kumar et al., (2018): Australia’s Health Star Rating policy process: Lessons for global policy-making in front-of-pack nutrition labelling 4. Mhurchu et al., (2017): Effects of a Voluntary Front-of-Pack Nutrition Labelling System on Packaged Food Reformulation: The Health Star Rating System in New Zealand 5. Morrison et al., (2018): Nutritional quality and reformulation of a selection of children's packaged foods available in Australian supermarkets: Has the Health Star Rating had an impact? 6. Hamlin et al., (2018) The Impact of the Australasian ‘Health Star Rating’, Front-of-Pack Nutritional Label, on Consumer Choice: A Longitudinal Study |
| Australian Food marketing policies | Food marketing | 1. Harker et al., (2008): Attributing Blame : Exploring the Link Between Fast Food Advertising and Obesity in Australia 2. Hawkes et al., (2011a): An analysis of the content of food industry pledges on marketing to children 3. Hawkes et al., (2011b): Regulating the commercial promotion of food to children: A survey of actions worldwide 4. Hebden et al., (2010a): Regulating the types of foods and beverages marketed to Australian children: How useful are food industry commitments? 5. Hebden et al., (2010): Industry self-regulation of food marketing to children: Reading the ﬁne print 6. Hebden et al., (2011): Advertising of fast food to children on Australian television: the impact of industry self-regulation 7. Jones et al., (2012): Branded food references in children's magazines: ‘advertisements’ are the tip of the iceberg 8. Jones et al., (2010): Children's magazines: reading resources or food marketing tools? 9. Kelly et al., (2007): Television food advertising to children: the extent and nature of exposure 10. King et al., (2011): Industry self-regulation of television food advertising: Responsible or responsive? 11. King et al., (2013): Building the case for independent monitoring of food advertising on Australian television 12. Reeve, (2011): The regulatory pyramid meets the food pyramid: Can regulatory theory improve controls on television food advertising to Australian children? 13. Reeve, (2013): Private Governance, Public Purpose? Assessing Transparency and Accountability in Self-Regulation of Food Advertising to Children 14. Reeve et al., (2018): Regulation of food advertising to children in six jurisdictions: a framework for analyzing and improving the performance of regulatory instruments 15. Russel et al., (2014): Determining the ‘healthiness’ of foods marketed to children on television using the Food Standards Australia New Zealand nutrient proﬁling criteria 16. Watson et al.., (2017): Advertising to children initiatives have not reduced unhealthy food advertising on Australian television 17. Roberts et al., (2012): Compliance with children’s television food advertising regulations in Australia 18. Roberts et al., (2014): Children’s exposure to food advertising: An analysis of the effectiveness of self-regulatory codes in Australia |
| Berkley SSB tax | Taxation | 1. Falbe et al., (2016): Impact of the Berkeley Excise Tax on Sugar-Sweetened Beverage Consumption 2. Hagenaars et al., (2017): The taxation of unhealthy energy-dense foods (EDFs) and sugar-sweetened beverages (SSBs): An overview of patterns observed in the policy content and policy context of 13 case studies 3. Lee et al., (2019): Sugar-Sweetened Beverage Consumption 3 Years After the Berkeley, California, Sugar-Sweetened Beverage Tax 4. Silver et al., (2017): Changes in prices, sales, consumer spending, and beverage consumption one year after a tax on sugar-sweetened beverages in Berkeley, California, US: A before-and-after study 5. World Cancer Research Fund International (2018). Building momentum: lessons on implementing a robust sugar sweetened beverage tax. |
| Brazil Voluntary sodium reformulation - | Sodium Reformulation | 1. Campbell et al., (2015): Inaugural Maximum Values for Sodium in Processed Food Products in the Americas 2. Nilson et al., (2017a): Sodium Reduction in Processed Foods in Brazil: Analysis of Food Categories and Voluntary Targets from 2011 to 2017 3. Nilson et al., (2017b): The impact of voluntary targets on the sodium content of processed foods in Brazil, 2011–2013 |
| Canada Sodium reformulation | Sodium Reformulation | 1. Campbell et al., (2014): Campbell et al., (2015): Inaugural Maximum Values for Sodium in Processed Food Products in the Americas 2. Campbell et al., (2011): Inaugural Maximum Values for Sodium in Processed Food Products in the Americas 3. Health Canada (2018): Sodium Reduction in processed foods in Canada: An evaluation of progress towards voluntary targets from 2012 to 2016 |
| Canadian Children’s Advertising initiative | Food marketing | 1. Kent et al., (2018):The effectiveness of self-regulation in limiting the advertising of unhealthy foods and beverages on children’s preferred websites in Canada 2. Kent et al., (2011): self-regulation by industry of food marketing is having little impact during children ’ s preferred television 3. Kent et al., (2014): Changes in the Volume, Power and Nutritional Quality of Foods Marketed to Children on Television in Canada 4. Kent et al., (2019): Food and beverage marketing in primary and secondary schools in Canada 5. Mulligan et al., (2018): Assessment of the Canadian Children’s Food and 6. Beverage Advertising Initiative’s Uniform Nutrition Criteria for Restricting Children’s Food and Beverage Marketing in Canada 7. Raine et al., (2013): Restricting marketing to children: Consensus on policy interventions to address obesity 8. Reeve et al., (2018): Regulation of food advertising to children in six jurisdictions: a framework for analyzing and improving the performance of regulatory instruments |
| Chile Food labelling and Advertising law | Labelling and food marketing | 1. Dintrans et al., (2020): Implementing a Food Labeling and Marketing Law in Chile 2. Reyes et al., (2020): Changes in the amount of nutrient of packaged foods and beverages after the initial implementation of the Chilean Law of Food Labelling and Advertising: A non-experimental prospective study 3. Reyes et al., (2019): Development of the Chilean front-of package food warning label 4. World Cancer Research Fund International (2019): Building momentum: lessons on implementing a robust front-of-pack food label 5. Correa et al., (2018): The prevalence and audience reach of food and beverage advertising on Chilean television according to marketing tactics and nutritional quality of products 6. Correa et al., (2020): Food Advertising on Television Before and After a National Unhealthy Food Marketing Regulation in Chile, 2016–2017 7. Carpentier et al., (2020): Evaluating the impact of Chile’s marketing regulation of unhealthy foods and beverages: preschool and adolescent children’s changes in exposure to food advertising on television |
| Danish Trans-fat Ban | Trans-fat reformulation | 1. Leth et al., (2006): The effect of the regulation on trans fatty acid content in Danish food 2. Restepo et al., (2016): Denmark’s Policy on Artiﬁcial Trans Fat and Cardiovascular Disease 3. Vallgårda (2018): The Danish trans-fatty acids ban: alliances, mental maps and co-production of policies and research |
| Danish whole grain logo | Labelling – Front of pack | 1. Greve et al., (2014): The Evolution of the Whole Grain Partnership in Denmark |
| Dutch choices Logo | Labelling – Front of pack | 1. Kelly et al., (2018): What is the evidence on the policy specifications, development processes and effectiveness of existing front-of-pack food labelling policies in the WHO European Region? 2. Vyth et al., (2009): A Front-of-Pack Nutrition Logo: A Quantitative and Qualitative Process Evaluation in the Netherlands 3. Smed et al., (2019): The effects of voluntary front-of-pack nutrition labels on volume shares of products: the case of the Dutch Choices 4. Jones et al., (2019): Front- of- pack nutrition labelling to promote healthier diets: current practice and opportunities to strengthen regulation worldwide |
| The Broadcasting Authority of Ireland (BAI) Code | Food marketing | 1. Reeve et al., (2018): Regulation of food advertising to children in six jurisdictions: a framework for analyzing and improving the performance of regulatory instruments 2. Tatlow-Golden et al., (2015): Creating good feelings about unhealthy food: children’s televised ‘advertised diet’ on the island of Ireland, in a climate of regulation |
| King Country Calorie labelling | Labelling – Menu Labelling | 1. Bruemmer et al., (2012): Energy, Saturated Fat, and Sodium Were Lower in Entrées at Chain Restaurants at 18 Months Compared with 6 Months Following the Implementation of Mandatory Menu Labeling Regulation in King County, Washington 2. Chen et al., (2015): Changes in Awareness and Use of Calorie Information After Mandatory Menu Labeling in Restaurants in King County, Washington 3. Finkelstein et al., (2011): Mandatory Menu Labeling in One Fast-Food Chain in King County, Washington 4. Johnson et al., (2012): Menu-Labeling Policy in King County, Washington 5. Krieger et al., (2013): Menu Labeling Regulations and Calories Purchased at Chain Restaurants 6. Saelens et al., (2012): Nutrition-Labeling Regulation Impacts on Restaurant Environments 7. Tandon et al., (2011): The Impact of Menu Labeling on Fast-Food Purchases for Children and Parents |
| New South Wales Calorie labelling | Labelling – New South Wales | 1. New South Wales Food Authority (2016): Evaluation of kilojoule menu labelling 2. Wellard-Cole et al., (2017): Monitoring the changes to the nutrient composition of fast foods following the introduction of menu labelling in New South Wales, Australia: an observational study 3. Wellard et al., (2015): The availability and accessibility of nutrition information in fast food outlets in five states post-menu labelling legislation in New South Wales |
| New York City Trans-fat Ban | Trans-fat reformulation | 1. Restepo et al., (2014): Trans Fat and Cardiovascular Disease Mortality: Evidence from Bans in Restaurants in New York 2. Sood et al., (2014): First Time Compliance Inspections to Evaluate an Artiﬁcial Trans Fat Ban in Nassau County 3. Tan (2009): A case study of the New York City trans-fat story for international application 4. Wright et al., (2019): impact of a municipal Policy restricting Trans Fatty Acid Use in New York in City Restaurants on Serum Trans Fatty Levels in Adult. |
| New York City Calorie labelling | Labelling | 1. Dumanovsky et al., (2010): Consumer Awareness of Fast-Food Calorie Information in New York City After Implementation of a Menu Labeling Regulation 2. Dumanovsky et al., (2010): Changes in energy content of lunchtime purchases from fast food restaurants after introduction of calorie labelling: cross sectional customer surveys 3. Elbel et al., (2009): Calorie Labeling And Food Choices: A First Look At The Effects On Low-Income People In NewYork City 4. Elbel et al., (2011): Child and adolescent fast-food choice and the influence of calorie labeling: a natural experiment 5. Farley et al., (2009): NewYork City’s Fight Over Calorie Labeling: A two-year struggle ultimately proves that innovation in food regulation is entirely possible at the local level. 6. Restrepo (2017): calorie labeling in chain restaurants and body weight: evidence from New York 7. Sisnowski (2016): Targeting population nutrition through municipal health and food policy: Implications of New York City’s experiences in regulatory obesity prevention |
| New Zealand | Food Marketing | 1. Field et al (2011): How do Vested Interests Maintain Outdated Policy? The Case of Food Marketing to New Zealand Children 2. Vandevijvere et al., (2017): Unhealthy food advertising directed to children on New Zealand television: extent, nature, impact and policy implications 3. Thornley et al., (2010): Does industry regulation of food advertising protect child rights? 4. Sing et al., (2020): Food Advertising to Children in New Zealand: A Critical Review of the Performance of a Self-Regulatory Complaints System Using a Public Health Law Framework |
| Philadelphia SSB tax | Taxation | 1. Hernández-F et al., (2019): Reduction in purchases of energy-dense nutrient-poor foods in Mexico associated with the introduction of a tax in 2014 2. Kane et al., (2019): Understanding beverage taxation: Perspective on the Philadelphia Beverage Tax’s novel approach 3. Roberto et al., (2019): Association of a Beverage Tax on Sugar-Sweetened and Artificially Sweetened Beverages with Changes in Beverage Prices and Sales at Chain Retailers in a Large Urban Setting. 4. World Cancer Research Fund International (2018). Building momentum: lessons on implementing a robust sugar sweetened beverage tax. 5. Zhong et al., (2018): The Short-Term Impacts of the Philadelphia Beverage Tax on Beverage Consumption |
| Quebec Consumer Protection Act | Food marketing | 1. Potvin Kent et al., (2013): Internet Marketing Directed at Children on Food and Restaurant Websites in Two Policy Environments 2. Raine et al., (2013): Restricting marketing to children: Consensus on policy interventions to address obesity 3. Reeve et al., (2018): Regulation of food advertising to children in six jurisdictions: a framework for analyzing and improving the performance of regulatory instrument |
| South African sodium maximum limit legislation | Sodium reformulation | 1. Charlton et al., (2014): To Legislate or Not to Legislate? A Comparison of the UK and South African Approaches to the Development and Implementation of Salt Reduction Programs 2. Kaldor et al. (2018): Using regulation to limit salt intake and prevent noncommunicable diseases: lessons from South Africa’s experience 3. Peters et al., (2017): The Sodium Content of Processed Foods in South Africa during the Introduction of Mandatory Sodium Limits 4. Swanepoel et al., (2017): Sodium content of foodstuﬀs included in the sodium reduction regulation of South Africa |
| South Korean Special Act on Safety Management of Children’s Dietary Life | Food marketing | 1. Kim et al., (2012): Restriction of television food advertising in South Korea: impact on advertising of food companies 2. Lee et al., (2013): effect of TV food advertising restriction on food environment for children in South Korea 3. Special act on safety management of children's dietary lifestyle - Act No. 8943, Mar. 21, 2008. |
| Spanish voluntary codes for food marketing | Food marketing | 1. Davó-Blanes et al., (2013): The impact of marketing practices and its regulation policies on childhood obesity. Opinions of stakeholders in Spain 2. Leon-Flandez et al., (2017): Evaluation of compliance with the Spanish Code of self-regulation of food and drinks advertising directed at children under the age of 12 years in Spain, 2012. 3. Ramos et al., (2015): Inﬂuence of Spanish TV commercials on child obesity 4. Romero-Ferna´ndez et al., (2009): Compliance with self-regulation of television food and beverage advertising aimed at children in Spain 5. Royo-Bordonada et al., (2016): The extent and nature of food advertising to children on Spanish television in 2012 using an international food-based coding system and the UK nutrient proﬁling model |
| Swedish food marketing regulations | Food marketing | 1. Caraher et al., (2005): Television advertising and children: lessons from policy development 2. Ó Cathaior (2017): Food Marketing to Children in Sweden and Denmark: a Missed Opportunity for Nordic Leadership 3. Sandberg (2011): Tiger talk and candy king: Marketing of unhealthy food and beverages to Swedish children |
| United Kingdom policies on food marketing |  | 1. Boyland et al., (2011): The extent of food advertising to children on UK television in 2008 2. Caraher et al., (2005): Television advertising and children: lessons from policy development 3. Garde et al., (2017): Case study: the UK rules on unhealthy food marketing to children 4. Landon, (2013): News report. Gaps and weaknesses in controls on food and drink marketing to children in the UK 5. Reeve et al., (2018): Regulation of food advertising to children in six jurisdictions: a framework for analyzing and improving the performance of regulatory instrument 6. Silva et al., (2015): An Evaluation of the Effect of Child-Directed Television Food Advertising Regulation in the United Kingdom. 7. Whalen et al., (2019): Children’s exposure to food advertising: the impact of Statutory regulations |
| United Kingdom Public Health responsibility deal | Sodium/Transfat reformulation | 1. Durand et al., (2015): An evaluation of the Public Health Responsibility Deal: Informants’ experiences and views of the development, implementation and achievements of a pledge-based, public–private partnership to improve population health in England 2. Knai et al., (2019): Has a public–private partnership resulted in action on healthier diets in England? An analysis of the Public Health Responsibility Deal food pledges 3. Laverty et al., (2019): Quantifying the impact of the Public Health Responsibility Deal on salt intake, cardiovascular disease and gastric cancer burdens: interrupted time series and microsimulation study 4. MacGregor et al., (2015): Quantifying the impact of the Public Health Responsibility Deal on salt intake, cardiovascular disease and gastric cancer burdens: interrupted time series and microsimulation study 5. Reeve & Magnusson (2015): Reprint of: Food reformulation and the (neo)-liberal state: new strategies for strengthening voluntary salt reduction programs in the UK and USA 6. Magnusson & Reeve (2015): Food Reformulation, Responsive Regulation, and “Regulatory Scaffolding”: Strengthening Performance of Salt Reduction Programs in Australia and the United Kingdom 7. Hutchson et al., (2017): Comparison of high and low trans-fatty acid consumers: analyses of UK National Diet and Nutrition Surveys before and after product reformulation |
| United Kingdom Sodium reformulation | Sodium reformulation | 1. Reeve & Magnusson (2015): Reprint of: Food reformulation and the (neo)-liberal state: new strategies for strengthening voluntary salt reduction programs in the UK and USA 2. MacGregor et al., (2015): Quantifying the impact of the Public Health Responsibility Deal on salt intake, cardiovascular disease and gastric cancer burdens: interrupted time series and microsimulation study 3. Magnusson & Reeve (2015): Food Reformulation, Responsive Regulation, and “Regulatory Scaffolding”: Strengthening Performance of Salt Reduction Programs in Australia and the United Kingdom 4. He et al., (2014): Salt reduction in the United Kingdom: a successful experiment in public health 5. Eyles et al., (2013): Impact of the UK voluntary sodium reduction targets on the sodium content of processed foods from 2006 to 2011: Analysis of household consumer panel data 6. Millet et al., (2012): Impacts of a National Strategy to Reduce Population Salt Intake in England: Serial Cross Sectional Study 7. Charlton et al., (2014): To Legislate or Not to Legislate? A Comparison of the UK and South African Approaches to the Development and Implementation of Salt Reduction Programs |
| United Kingdom Soft Drinks Levy | Taxation | 1. Buckton et al., (2019): A discourse network analysis of UK newspaper coverage of the “sugar tax” debate before and after the announcement of the Soft Drinks Industry Levy 2. Hilton et al., (2019): Following in the footsteps of tobacco and alcohol? Stakeholder discourse in UK newspaper coverage of the Soft Drinks Industry Levy 3. Bandy et al., (2020): Reductions in sugar sales from soft drinks in the UK from 2015 to 2018 |
| United States National Salt reduction initiative | Reformulation | 1. Curtis et al., (2016): US Food Industry Progress During the National Salt Reduction Initiative: 2009–2014 2. Hennie et al., (2009): Strategies to reduce sodium intake in the United States 3. Reeve & Magnusson (2015): Reprint of: Food reformulation and the (neo)-liberal state: new strategies for strengthening voluntary salt reduction programs in the UK and USA |
| United Children’s Food and Beverage Advertising Initiative | Food Marketing | 1. Bernhardt et al., (2013): How Television Fast Food Marketing Aimed at Children Compares with Adult Advertisements 2. Harris et al., (2015): Sweet promises: Candy advertising to children and implications for industry self-regulation 3. Kunkel et al., (2013) : Food Marketing to Children on U.S. Spanish Language Television 4. Kunkel et al., (2014): Solution or Smokescreen? Evaluating Industry Self-Regulation of Televised Food Marketing to Children 5. Kunkel et al., (2015): Evaluating Industry Self-Regulation of Food Marketing to Children 6. Dietz (2013): New Strategies To Improve Food Marketing To Children 7. Hoy et al., (2015): The evolution of self-regulation in food advertising 8. Mello, (2010): Federal Trade Commission Regulation of Food Advertising to Children: Possibilities for a Reinvigorated Role 9. Kraak et al., (2014): An accountability evaluation for the industry’s responsible use of brand mascots and licensed media characters to market a healthy diet to American children 10. Powell et al., (2010): Trends in Exposure to Television Food Advertisements Among Children and Adolescents in the United States 11. Powell et al., (2010): Trends in the Nutritional Content of Television Food Advertisements Seen by Children in the United States 12. Powell et al., (2013): Nutritional Content of Food and Beverage Products in Television Advertisements Seen on Children’s Programming 13. Reeve et al., (2018): Regulation of food advertising to children in six jurisdictions: a framework for analyzing and improving the performance of regulatory instrument 14. Salinsky, (2006): Effects of Food Marketing to Kids: I’m Lovin’ It? 15. Schermbeck (2015): Nutrition Recommendations and the Children’s Food and Beverage Advertising Initiative’s 2014 Approved Food and Beverage Product List 16. Schwarz et al., (2012): Food Marketing to Youth: Current Threats and Opportunities 17. Wootan et al., (2019): How Do Nutrition Guidelines Compare for Industry to Market Food and Beverage Products to Children? World Health Organization Nutrient Proﬁle Standards versus the US Children’s Food and Beverage Advertising Initiative |
