# Supplementary material for: The regulatory governance conditions that lead to food policies achieving improvements in population nutrition outcomes: a qualitative comparative analysis
Source: Public Health Nutr. 2021 Dec 7;25(5):1395–405. doi: 10.1017/S1368980021004730 (PMC9991667; doi:10.1017/S1368980021004730)
Supplement: Supplementary file 1 [file S1368980021004730sup.zip › S1368980021004730sup004.docx]

*Supplementary file 4: Data Table*

| Policy Cases | Industry Involvement | Regulatory Design | Instrument Design | Monitoring | Enforcement | Outcomes |
| --- | --- | --- | --- | --- | --- | --- |
| Australian health star rating | 0.66 | 0.33 | 0.33 | 1 | 0.33 | 1 |
| Australian food marketing | 1 | 0 | 0 | 0.33 | 0 | 0 |
| Australian food & health dialogue | 0.66 | 0.33 | 0.33 | 0.33 | 0 | 1 |
| Brazil sodium reformulation | 0.66 | 0.33 | 1 | 1 | 0 | 1 |
| Berkeley SSB tax | 0 | 1 | 0.66 | 1 | 1 | 1 |
| Chile Front of pack warning labels | 0 | 1 | 1 | 1 | 1 | 1 |
| Canadian Children’s advertising initiative | 1 | 0 | 0 | 0.33 | 0 | 0 |
| Canadian sodium reformulation | 0.66 | 0.33 | 1 | 1 | 0 | 0 |
| Danish wholegrain logo | 0.66 | 0.33 | 1 | 0.66 | 0 | 1 |
| Denmark trans-fat ban | 0.33 | 1 | 1 | 1 | 1 | 1 |
| Dutch choices logo | 0.66 | 0.33 | 1 | 1 | 0 | 1 |
| Irish Broadcasting Code | 0 | 1 | 1 | 1 | 1 | 1 |
| King County Calorie labelling | 0 | 1 | 0.66 | 1 | 0.66 | 1 |
| New York City Trans-fat ban | 0.33 | 1 | 0.66 | 1 | 1 | 1 |
| New Zealand food marketing | 1 | 0 | 0.33 | 0.33 | 0 | 0 |
| Philadelphia SSB tax | 0 | 1 | 1 | 1 | 1 | 1 |
| Quebec | 0 | 1 | 0.33 | 0.33 | 0.66 | 0 |
| South African Sodium reformulation | 0.33 | 1 | 1 | 1 | 1 | 1 |
| South Korea Food Marketing regulations | 0 | 1 | 0.66 | 1 | 1 | 1 |
| Spanish food marketing regulations | 0.66 | 0 | 0 | 1 | 0.66 | 0 |
| Swedish Food Marketing regulations | 0 | 1 | 0.33 | 0 | 0.66 | 0 |
| United Kingdom Soft Drinks Industry Levy | 0 | 1 | 0.66 | 1 | 1 | 1 |
| United Kingdom sodium reformulation (Food Standards Agency) | 0.33 | 0.33 | 1 | 1 | 0 | 1 |
| United Kingdom sodium reformulation (Public Health responsibility deal) | 0.66 | 0.33 | 0.66 | 0.33 | 0 | 0 |
| Policy Case | **Industry involvement** | **Regulatory design** | **Instrument design** | **Monitoring** | **Enforcement** | **Outcomes** |
| United States National Sodium Reduction Initiative | 0.33 | 0.33 | 1 | 1 | 0 | 1 |
| United States Children’s Food and Beverage Advertising Initiative | 1 | 0 | 0 | 0.33 | 0 | 0 |
